# Spontaneous brain activity in the hippocampal regions could characterize cognitive impairment in patients with Parkinson's disease

**DOI:** 10.1111/cns.14706

**Published:** 2024-04-07

**Authors:** Peng Chen, Guoqiang Tang, Yanglingxi Wang, Weiming Xiong, Yongbing Deng, She Fei, Jun Zhang

**Affiliations:** ^1^ Department of Neurosurgery, Chongqing Key Laboratory of Emergency Medicine, Chongqing Emergency Medical Center Chongqing University Central Hospital Chongqing China; ^2^ Pre‐hospital Emergency Department, Chongqing Emergency Medical Center Chongqing University Central Hospital Chongqing China; ^3^ Department of Emergency The Fourth Medical Center of the Chinese PLA General Hospital Beijing China; ^4^ Department of Neurosurgery, Huashan Hospital, Shanghai Medical College Fudan University Shanghai China; ^5^ Department of Neurosurgery Clinical Medical College of Yangzhou University Yangzhou China

**Keywords:** cognitive impairment, fractional amplitude of low‐frequency fluctuation, hippocampal regions; spontaneous brain activity, Parkinson's disease

## Abstract

**Objective:**

This study aimed to investigate whether spontaneous brain activity can be used as a prospective indicator to identify cognitive impairment in patients with Parkinson's disease (PD).

**Methods:**

Resting‐state functional magnetic resonance imaging (RS‐fMRI) was performed on PD patients. The cognitive level of patients was assessed by the Montreal Cognitive Assessment (MoCA) scale. The fractional amplitude of low‐frequency fluctuation (fALFF) was applied to measure the strength of spontaneous brain activity. Correlation analysis and between‐group comparisons of fMRI data were conducted using Rest 1.8. By overlaying cognitively characterized brain regions and defining regions of interest (ROIs) based on their spatial distribution for subsequent cognitive stratification studies.

**Results:**

A total of 58 PD patients were enrolled in this study. They were divided into three groups: normal cognition (NC) group (27 patients, average MoCA was 27.96), mild cognitive impairment (MCI) group (21 patients, average MoCA was 23.52), and severe cognitive impairment (SCI) group (10 patients, average MoCA was 17.3). It is noteworthy to mention that those within the SCI group exhibited the most advanced chronological age, with an average of 74.4 years, whereas the MCI group displayed a higher prevalence of male participants at 85.7%. It was found hippocampal regions were a stable representative brain region of cognition according to the correlation analysis between the fALFF of the whole brain and cognition, and the comparison of fALFF between different cognitive groups. The parahippocampal gyrus was the only region with statistically significant differences in fALFF among the three cognitive groups, and it was also the only brain region to identify MCI from NC, with an AUC of 0.673. The paracentral lobule, postcentral gyrus was the region that identified SCI from NC, with an AUC of 0.941. The midbrain, hippocampus, and parahippocampa gyrus was the region that identified SCI from MCI, with an AUC of 0.926.

**Conclusion:**

The parahippocampal gyrus was the potential brain region for recognizing cognitive impairment in PD, specifically for identifying MCI. Thus, the fALFF of parahippocampal gyrus is expected to contribute to future study as a multimodal fingerprint for early warning.

## Graphical abstract

Spontaneous brain activity (fractional amplitude of low‐frequency fluctuation − fALFF) in the hippocampal regions could reveal cognitive levels in patients with Parkinson's disease to differentiate normal cognition, mild cognitive impairment, and severe cognitive impairment.

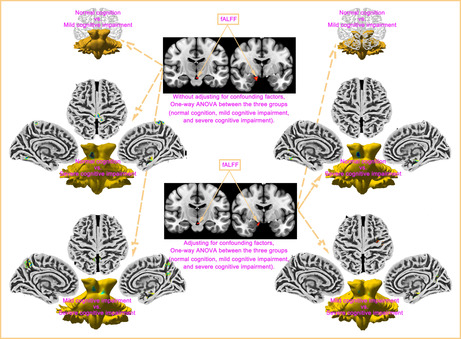

## 1 INTRODUCTION

Parkinson's disease (PD) is a clinically common neurodegenerative disease characterized by motor symptoms, including tremors, bradykinesia, and rigidity. In addition to motor symptoms, non‐motor symptoms are considered to precede motor symptoms, affecting the quality of life.^1^, ^2^ Common non‐motor symptoms of PD include dementia, neuropsychiatric symptoms, autonomic failure, and sensory impairments. Dementia is most detrimental to the quality of life and increases mortality rates.^3^, ^4^ Mild cognitive impairment (MCI) is considered a transitional stage between normal state and full‐blown dementia and is also a powerful predictor of dementia.^5^, ^6^

Currently, the diagnostic criteria for PD‐MCI still need further revision and refinement.^5^, ^7^, ^8^, ^9^ Therefore, the research on PD‐MCI is increasing rapidly, and exploring the characteristics of different cognitive states in PD and the laws of transition between states is the focus of disease pathology research, especially finding the predictors of PD‐MCI to provide the theoretical reference for early treatment and rehabilitation.

The research found that demographic indicators of gender were a critical factor, that men were more likely to develop cognitive impairment, and that characteristics of cognitive impairment differed between genders.^10^ Other factors included genotype, in which the Catechol‐O‐methyltransferase genotype was related to executive‐attention function^11^, ^12^ and the apolipoprotein E genotype was related to cognitive decline.^13^

In the study of radiological imaging predictive factors, the cross‐sectional analysis suggested that PD‐MCI was more likely to develop brain atrophy, occurring in areas such as the left superior frontal gyrus, superior temporal lobe, insula,^14^ right supramarginal gyrus, bilateral dorsolateral prefrontal cortex, and midcingulate cortex.^15^ Cerebral small vessel disease and its imaging characteristics were related to gait, and cognition in PD patients. It was found white matter hyperintensity was associated with slow gait speed, decreased cadence, increased stride time, and increased stance phase time. The presence of lacune was associated with poor attention and impaired executive function. It was demonstrated white matter hyperintensity, number of lacunes, and microbleeds were positively correlated with the severity of motor, cognitive, and emotional impairments, while the perivascular space in the basal ganglia was only correlated with cognitive impairments. Moreover, monitoring cerebral blood flow by arterial spin labeling (ASL)‐MRI, researchers found hypoperfusion was a predictor of cognitive impairment in PD patients.^16^, ^17^ The above studies revealed multidimensional imaging markers of comorbid cognitive impairment in PD. However, the relationship between spontaneous brain activity and comorbid cognitive impairment in PD is unclear.

The emerging resting‐state functional magnetic resonance (RS‐fMRI) technology has brought new methods for collecting brain neural activity. With the development of methodology, the proposal of fractional amplitude of low‐frequency fluctuations (fALFF) laid the foundation for characterizing neural activity. fALFF can be applied to analyze brain activity in the state of neurological diseases is widely used to explore the mechanism of neurological diseases, and has been found to have significant repeatability. In conclusion, this study used the RS‐fMRI method to detect the brain activity by fALFF in the cognitive brain regions in PD patients, and innovatively found the characteristics of cognitive stratification by comparing the differences in spontaneous brain activity between different cognitive groups.

## 2 METHODS

### 2.1 Participants

The fMRI data were obtained from the brain imaging shared database − OpenNeuro (https://openneuro.org/), with dataset number ds004392. This was a resting state functional imaging dataset comprising PD patients with different cognitive levels. In addition, the data also included baseline information such as cognitive scores (Montreal Cognitive Assessment, MoCA), age, gender, handedness, and education level. The dataset included patients with the following features: (1) Patients diagnosed with PD; (2) Age: 55–89 years; (3) Patients with different cognitive levels: MoCA 9–30; (4) years of education level: 7–24 years.

### 2.2 Cognitive level categorization

The MoCA was primarily utilized for evaluating cognitive function in elderly individuals and patients with neurological disorders by assessing attention, memory, language, and spatial abilities, with a total score of 30 points.^18^ MoCA played a crucial role in the early detection of cognitive impairments, diagnosis of MCI, and evaluation of treatment efficacy. According to the distribution of MoCA, cognitive levels were classified into NC group (MoCA > 25), MCI group (MoCA 22–24), and severe cognitive impairment (SCI) group (MoCA < 22).^19^, ^20^, ^21^, ^22^ Furthermore, MCI together with SCI groups is considered to be the cognitively abnormal group.

### 2.3 Parameters of RS‐fMRI

RS‐fMRI scans were performed by using a 3.0 Tesla system (GE Medical Systems, ModelName: Signa HDxt) with gradient echo planar (percent phase field view = 100, flip angle = 70°, echo time = 28 ms, repetition time = 2000 ms, total readout time: 32.76 ms, pixel bandwidth = 7812.5; matrix = 64 × 64, slice thickness = 3.5 mm, and spacing between slices 4 mm).

In addition, a 3D T1‐weighted image was acquired covering the whole brain (percent phase field view = 100, flip angle =10°, echo time = 2.988 ms, repetition time = 10.02 ms, pixel bandwidth = 122.109, matrix = 256 × 256, slice thickness = 1 mm, and spacing between slices = 1).

### 2.4 Image data pre‐processing

The MATLAB 2016b software with the Statistical Parametric Mapping (SPM 12, http://www.fil.ion.ucl.ac.uk/spm) package was used for pre‐processing the resting state fMRI data. The first 10 volumes were discarded to eliminate the machine instability interference.^23^ In further processing, slice timing, head motion correction (Friston 24), segment, spatial normalization to a Montreal Neurological Institute (MNI) template (resampling voxel size = 3 mm × 3 mm × 3 mm), smoothing (Gauss kernel of 4 mm with full width and half height), and linear trend removal were performed.^23^

### 2.5 fMRI data post‐processing

Rest 1.8 (http://www.restfmri.net/forum/REST_V1.8) software was applied for further processing. The time series of each voxel was transformed into the frequency domain and a power spectrum was then obtained. The square root of the power spectrum was calculated at each frequency and the average square root of the power spectrum within the frequency band of 0.01–0.08 Hz was retained as the amplitude of low‐frequency fluctuation (ALFF).^24^ Additionally, fALFF was defined as the ratio of the power of each frequency at the low‐frequency range (0.01–0.08 Hz) to that of the entire detectable frequency range (0–0.25 Hz).^25^ The fALFF could accurately reflect the amplitude levels of brain regions within a specific frequency band, reducing inter‐individual differences and the influence of global signals.^25^ Simultaneously, fALFF mitigates interference from physiological sources like heartbeat and respiration as it normalizes the amplitude for every frequency band.^25^ Therefore, fALFF was selected to enhance data reliability and interpretability to investigate the correlation between spontaneous brain activity and cognition.

The impact of confounding variables on the outcomes was also elucidated in subsequent analyses, predominantly by adjusting for confounding variables (such as age, gender, handedness, education level) versus not making any adjustments. First, voxel‐based correlation analysis was conducted to investigate the association between fALFF and MoCA to identify brain regions associated with cognitive impairment (AlphaSim corrected *p* < 0.01, cluster size >19 voxels). Second, one‐way ANOVA and post hoc analyses were applied to compare the differences in brain activity between three cognitive groups (AlphaSim corrected *p* < 0.01, cluster size >19 voxels). Third, based on the prior knowledge of hippocampus and cognition, we performed correlation analysis between fALFF and MoCA for hippocampal regions to further screen cognitive representative regions of patients with PD. Fourth, a two‐sample *t*‐test was used to compare the differences in brain activity in the hippocampal region between the cognitively abnormal and the NC groups. Considering the smaller voxel levels in the hippocampal region, we used a relatively loose correction (AlphaSim corrected *p* < 0.01, cluster size >1 voxel) aimed at improving the sensitivity of the results. Subsequently, Anchored neurocognitive brain regions from the first three steps were overlapped to generate independent regions of interest (ROI) based on their spatial distribution in a sequential manner. Finally, the fALFF between the three cognitive groups was extracted, based on ROIs from overall and triple group level, for subsequent between‐group identification.

### 2.6 Routine statistical analyses

The statistical analysis and graphical representation were conducted using SPSS 26 (https://www.ibm.com/products/spss‐statistics), MedCalc (https://www.medcalcsoftware.com/), and GraphPad Prism 9.5 (https://www.graphpad.com/). Initially, the normality of the continuous variables was assessed through the Kolmogorov–Smirnov test (SPSS 26). If the distribution was found to be normal, inter‐group comparisons were performed using two‐sample *t*‐test or one‐way analysis of variance with post‐hoc pair‐wise comparisons, reported as mean ± standard deviation (SPSS 26 and GraphPad Prism 9.5). In cases of non‐normal distribution, the Kruskal‐Wallis test was utilized, and the results were presented as median and quartiles (SPSS 26). The count data were compared using the Chi‐square test (SPSS 26).

Receiver operating characteristic (ROC) curves were utilized for constructing a predictive measure (MedCalc). The discriminative capability of fALFF was assessed by computing the area under the ROC curve (AUC). An AUC value of 1.0 denotes perfect discrimination, while an AUC value of 0.5 implies a lack of discriminative ability.

## 3 RESULTS

### 3.1 Demographic analysis

A total of 58 PD patients were enrolled in this study, with an average age of 70.03 years, predominantly male, accounting for 67.2% (39 patients), and predominantly right‐handed, accounting for 87.9% (51 patients). The education level was 16.21 years, and the average cognitive score (MoCA) was 24.52. According to the MoCA, patients were divided into three groups: NC group (27 patients, average MoCA was 27.96), MCI group (21 patients, average MoCA was 23.52), and SCI group (10 patients, average MoCA was 17.3). Patients with SCI were the oldest at 74.4 years in three cognitive groups. In terms of gender, patients with MCI had a higher proportion of males (85.7%) than other groups. There was no significant difference in the education level and the proportion of right‐handed in the three cognitive groups (Table 1).

**TABLE 1:** Baseline characteristics of patients with PD.

Characteristics	All patients	Normal cognition	Mild cognitive impairment	Severe cognitive impairment	*p*‐value
Age (mean ± SD)	70.03 ± 7.84	67.85 ± 7.59	70.76 ± 6.53	74.40 ± 9.56	0.19^a^, 0.02^b^, 0.22^c^
Gender, male (%)	39/58 (67.2)	13/27 (48.2)	18/21 (85.7)	8/10 (80.0)	0.02^a^, 0.17^b^, 1.00^c^
Right‐handed, *N* (%)	51/58 (87.9)	25/27 (92.6)	16/21 (76.2)	8/10 (80.0)	0.24^a^, 0.62^b^, 1.00^c^
Education (years, mean ± SD)	16.21 ± 2.75	16.37 ± 2.48	16.33 ± 2.31	15.50 ± 4.20	0.96^a^, 0.40^b^, 0.44^c^
MoCA, (mean ± SD)	24.52 ± 4.26	27.96 ± 1.40	23.52 ± 1.03	17.30 ± 3.34	<0.001^a,b,c^

*Note*: *N*, number (one‐way analysis of variance with post‐hoc pair‐wise comparisons or Chi‐square test: ^a^mild cognitive impairment group vs. normal cognition group; ^b^severe cognitive impairment group vs. normal cognition group; ^c^severe cognitive impairment group vs. mild cognitive impairment group).

Abbreviation: SD, standard deviation.

### 3.2 Correlation analysis between fALFF of whole brain and cognition in PD patients

**FIGURE 1:**
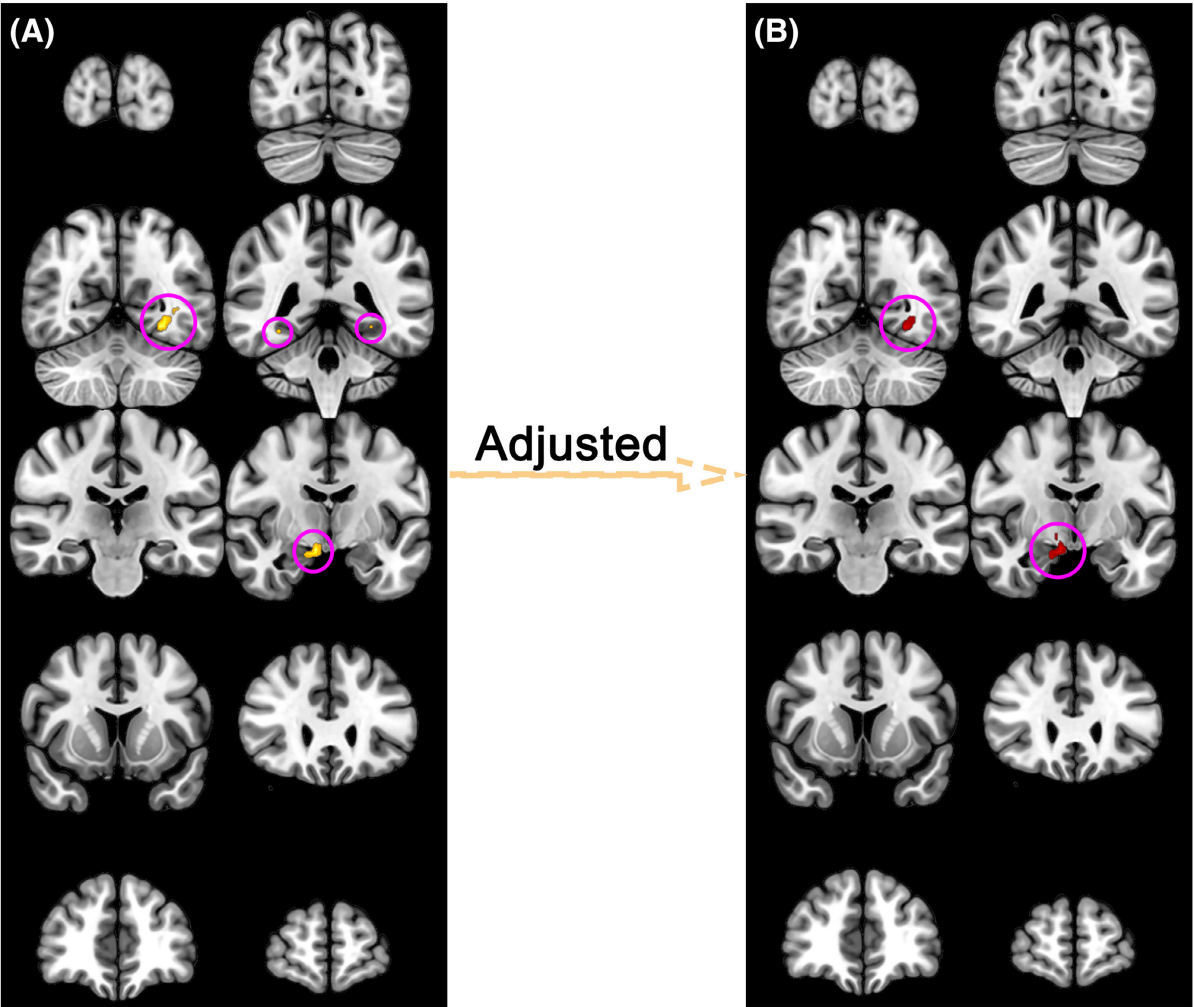
Correlation between whole brain fALFF level and MoCA in PD patients. (A) Without adjusting for confounding factors, brain regions correlated with cognition included the Right Brainstem, Midbrain, Limbic Lobe, Hippocampus, Parahippocampal Gyrus, Amygdala, Fusiform, Occipital Lobe, Parahippocampal Gyrus, Lingual Gyrus, Middle Temporal Gyrus. (B) After adjusting for confounding factors, brain regions associated with cognition included Right Brainstem, Midbrain, Limbic Lobe, Hippocampus, Parahippocampal Gyrus, Amygdala, Occipital Lobe, Fusiform Gyrus, Parahippocampal Gyrus, Temporal Lobe, Lingual Gyrus, Posterior Cingulate.

We analyzed the correlation between whole brain fALFF by voxels and MoCA in PD patients. Without adjusting for confounding factors such as age, gender, handedness, education level, brain regions correlated with cognition included Right Brainstem, Midbrain, Limbic Lobe, Hippocampus, Parahippocampa Gyrus, Amygdala, Fusiform, Occipital Lobe, Parahippocampa Gyrus, Lingual Gyrus, Middle Temporal Gyrus (Figure 1A). After adjusting for confounding factors, brain regions associated with cognition included Right Brainstem, Midbrain, Limbic Lobe, Hippocampus, Parahippocampa Gyrus, Amygdala, Occipital Lobe, Fusiform Gyrus, Parahippocampa Gyrus, Temporal Lobe, Lingual Gyrus, Posterior Cingulate (Figure 1B). We found that after adjusting for confounding factors, only the posterior cingulate was newly identified as a cognitive‐related brain region (Table 2).

**TABLE 2:** Correlation between fALFF at the voxel level across the whole brain in patients with PD and MoCA scores.

Voxels	Regions	Peak MNI	Peak intensity	_AlphaSim*_*_ *p* value
		*x*/*y*/*z*		
Non‐adjusted				
22	Right Brainstem, Midbrain, Limbic Lobe, Hippocampus, Parahippocampa Gyrus, Amygdala	9/−9/−15	0.53	<0.01
38	Fusiform, Occipital Lobe, Limbic Lobe, Parahippocampa Gyrus, Lingual Gyrus, Middle Temporal Gyrus	−27/−57/−9	0.50	<0.01
25	Fusiform, Parahippocampa Gyrus, Limbic Lobe, Occipital Lobe, Lingual Gyrus	27/−51/−9	0.52	<0.01
Adjusted				
24	Right Brainstem, Midbrain, Limbic Lobe, Hippocampus, Parahippocampa Gyrus, Amygdala	9/−9/−15	0.54	<0.01
33	Occipital Lobe, Fusiform Gyrus, Limbic Lobe, Parahippocampa Gyrus, Temporal Lobe, Lingual Gyrus, Posterior Cingulate	−27/−60/−9	0.51	<0.01

Abbreviation: MNI, montreal neurological institute.

### 3.3 Comparison of fALFF between different cognitive groups

**FIGURE 2:**
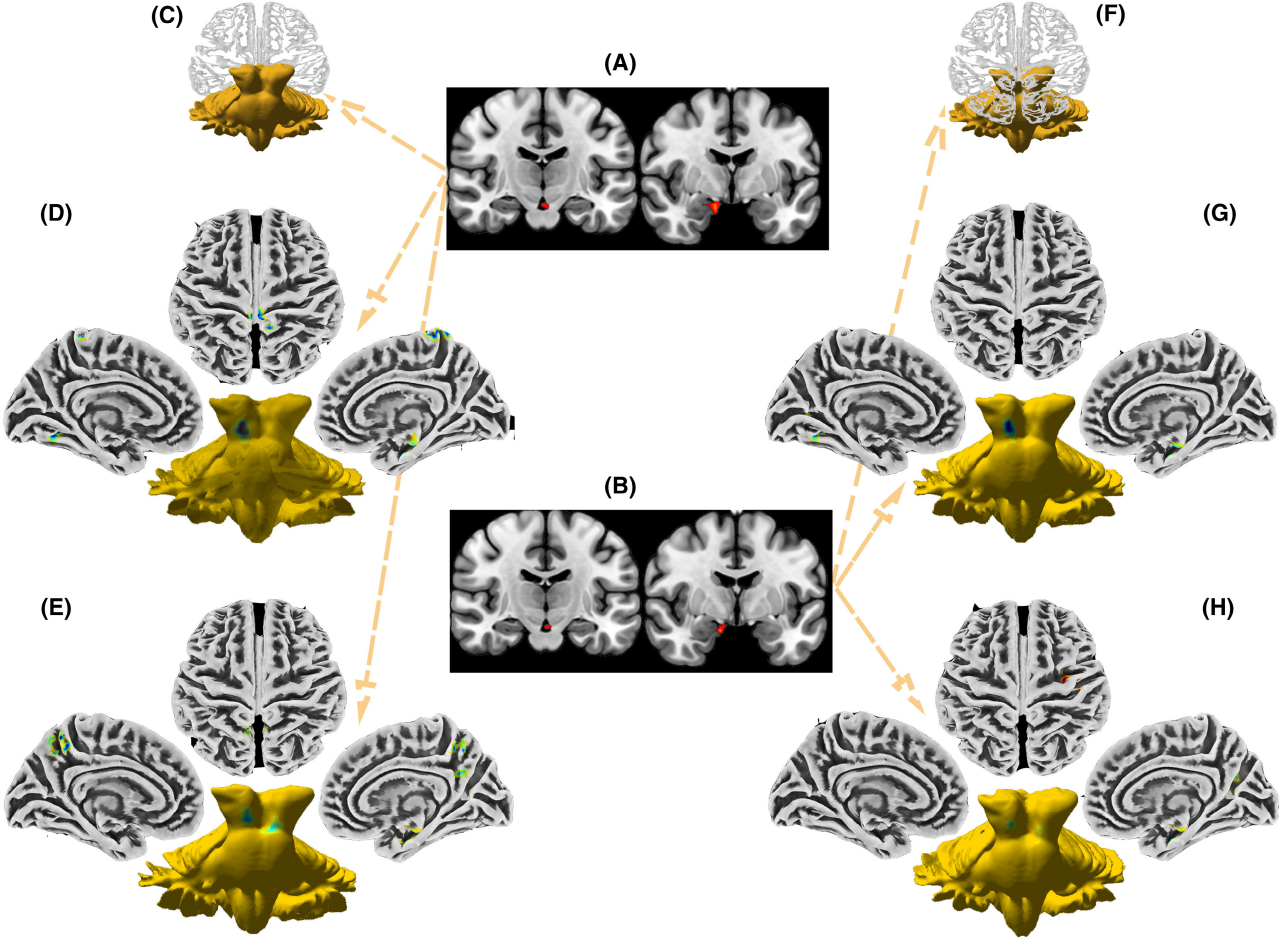
Comparison of fALFF between three cognitive groups. (A) Without adjusting for confounding factors, brain regions with differences in fALFF among three cognitive groups included Midbrain, Limbic Lobe, Parahippocampal Gyrus, Brainstem, Brodmann area 34, Amygdala, Frontal Lobe. (B) After adjusting for confounding factors, brain regions with differences in fALFF among three cognitive groups included Midbrain, Brainstem, Parahippocampal Gyrus, Limbic Lobe, Amygdala, Brodmann area 34, and Frontal Lobe. brain regions among the three cognitive groups were basically the same, whether the confounding factors were adjusted or not. (C, F) There was no statistically significant difference in fALFF between PD patients with MCI and NC, regardless of adjustment for confounding factors. (D) Comparing PD patients with SCI and NC, without adjusting for confounding factors, there were differences in fALFF including Brainstem, Midbrain, Parahippocampal Gyrus, Brodmann area 34, Limbic Lobe, Mammillary body, Hippocampus, Occipital Lobe, Precuneus, Frontal Lobe, Brodmann area 4, Paracentral Lobule, Postcentral Gyrus. (G) Comparing PD patients with SCI and NC, after adjusting for confounding factors, there were differences in fALFF including Brainstem, Midbrain, Parahippocampal Gyrus, Limbic Lobe, Brodmann area 34, Occipital Lobe. (E) Comparing PD patients with SCI and MCI, without adjusting for confounding factors, there were differences in fALFF including Parahippocampal Gyrus, Limbic Lobe, Midbrain, Brodmann area 34, Brainstem, Amygdala, Frontal Lobe, Hippocampus, Precuneus, Brodmann area 31, Posterior Cingulate, Brodmann area 7. (H) Comparing PD patients with SCI and MCI, after adjusting for confounding factors, there were differences in fALFF including Midbrain, Brainstem, Limbic Lobe, Parahippocampal Gyrus, Brodmann area 34, Precuneus, Calcarine, Brodmann area 31, Occipital Lobe, Posterior Cingulate, Precentral Gyrus, Frontal Lobe.

One‐way ANOVA was used to compare the fALFF in three cognitive groups. Without adjusting for confounding factors such as age, gender, handedness, education level, brain regions with differences in fALFF among three cognitive groups included Midbrain, Limbic Lobe, Parahippocampal Gyrus, Brainstem, Brodmann area 34, Amygdala, Frontal Lobe (Figure 2A, Table S1). After adjusting for confounding factors, brain regions with differences in fALFF among three cognitive groups included the Midbrain, Brainstem, Parahippocampal Gyrus, Limbic Lobe, Amygdala, Brodmann area 34, and Frontal Lobe (Figure 2B, Table S2). We found that whether the confounding factors were adjusted or not, differential brain regions among the three cognitive groups were basically the same, but the voxel number values of the brain regions were different.

We further compared the differences between the three cognitive groups with each other. We found no statistically significant difference in fALFF between PD patients with MCI and NC, regardless of adjustment for confounding factors (Figure 2C,F).

When comparing PD patients with SCI and NC, without adjusting for confounding factors, there were differences in fALFF including Brainstem, Midbrain, Parahippocampal Gyrus, Brodmann area 34, Limbic Lobe, Mammillary Bod, Hippocampus, Occipital Lobe, Precuneus, Frontal Lobe, Brodmann area 4, Paracentral Lobule, Postcentral Gyrus (Figure 2D, Table S3). After adjusting for confounding factors, the different brain regions were further converged, including differences in fALFF of the Brainstem, Midbrain, Parahippocampal Gyrus, Limbic Lobe, Brodmann area 34, Occipital Lobe (Figure 2G, Table S4).

When comparing PD patients with SCI and MCI, without adjusting for confounding factors, there were differences in fALFF including Parahippocampa Gyrus, Limbic Lobe, Midbrain, Brodmann area 34, Brainstem, Amygdala, Frontal Lobe, Hippocampus, Precuneus, Brodmann area 31, Posterior Cingulate, brodmann area 7 (Figure 2E, Table S5). After adjusting for confounding factors, differential brain regions were basically the same, but the voxel number values of the brain regions were decreased, including differences in fALFF of Midbrain, Brainstem, Limbic Lobe, Parahippocampa Gyrus, Brodmann area 34, Precuneus, Calcarine, Brodmann area 31, Occipital Lobe, Posterior Cingulate, Precentral Gyrus, Frontal Lobe (Figure 2H, Table S6).

### 3.4 Correlation analysis between fALFF of hippocampus and cognition in PD patients

**FIGURE 3:**
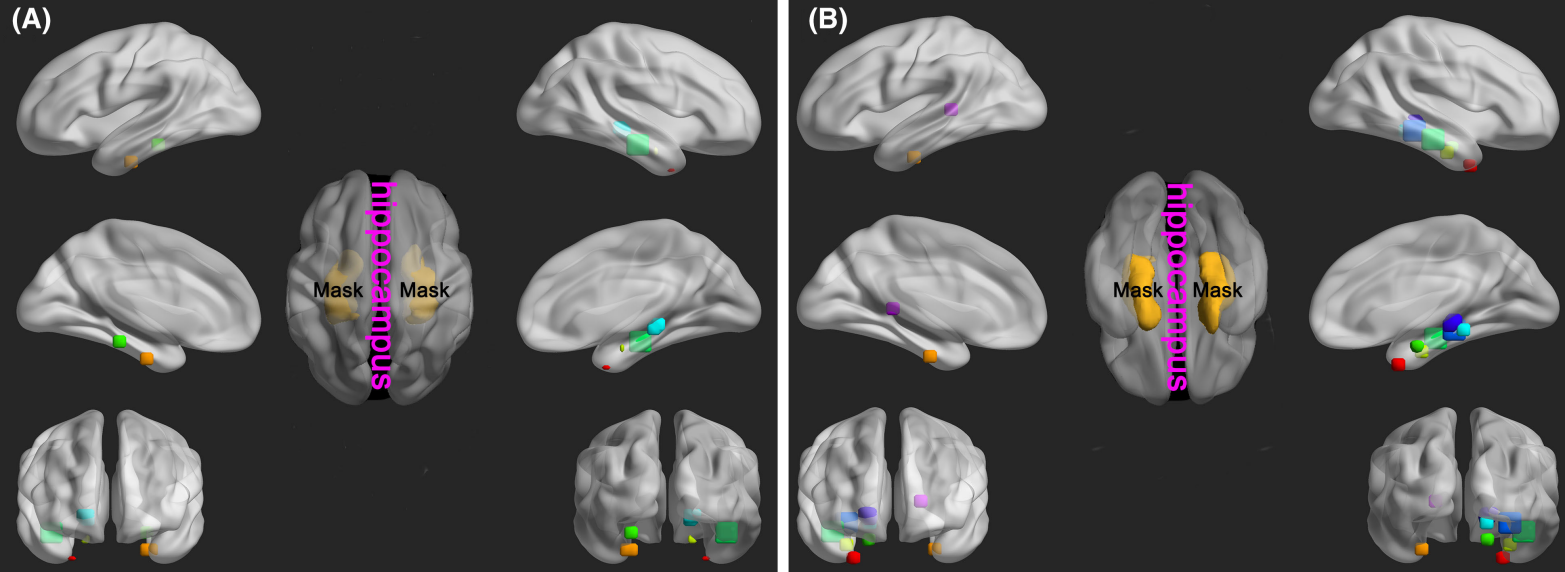
Correlation between hippocampus fALFF level and MoCA in PD patients. (A) The hippocampus was a stable representative brain region of cognition. Related regions in the hippocampus without adjusting for confounding factors. (B) The hippocampus was a stable representative brain region of cognition. Related regions in the hippocampus after adjusting confounding factors.

Based on the prior knowledge of hippocampus and cognition, we further analyzed the correlation between hippocampus fALFF by voxels and MoCA in PD patients. We found the hippocampus was a stable representative brain region of cognition, regardless of adjustment for confounding factors (Figure 3, Tables S7 and S8).

### 3.5 Differences in hippocampal fALFF between the cognitively abnormal and the NC groups

**FIGURE 4:**
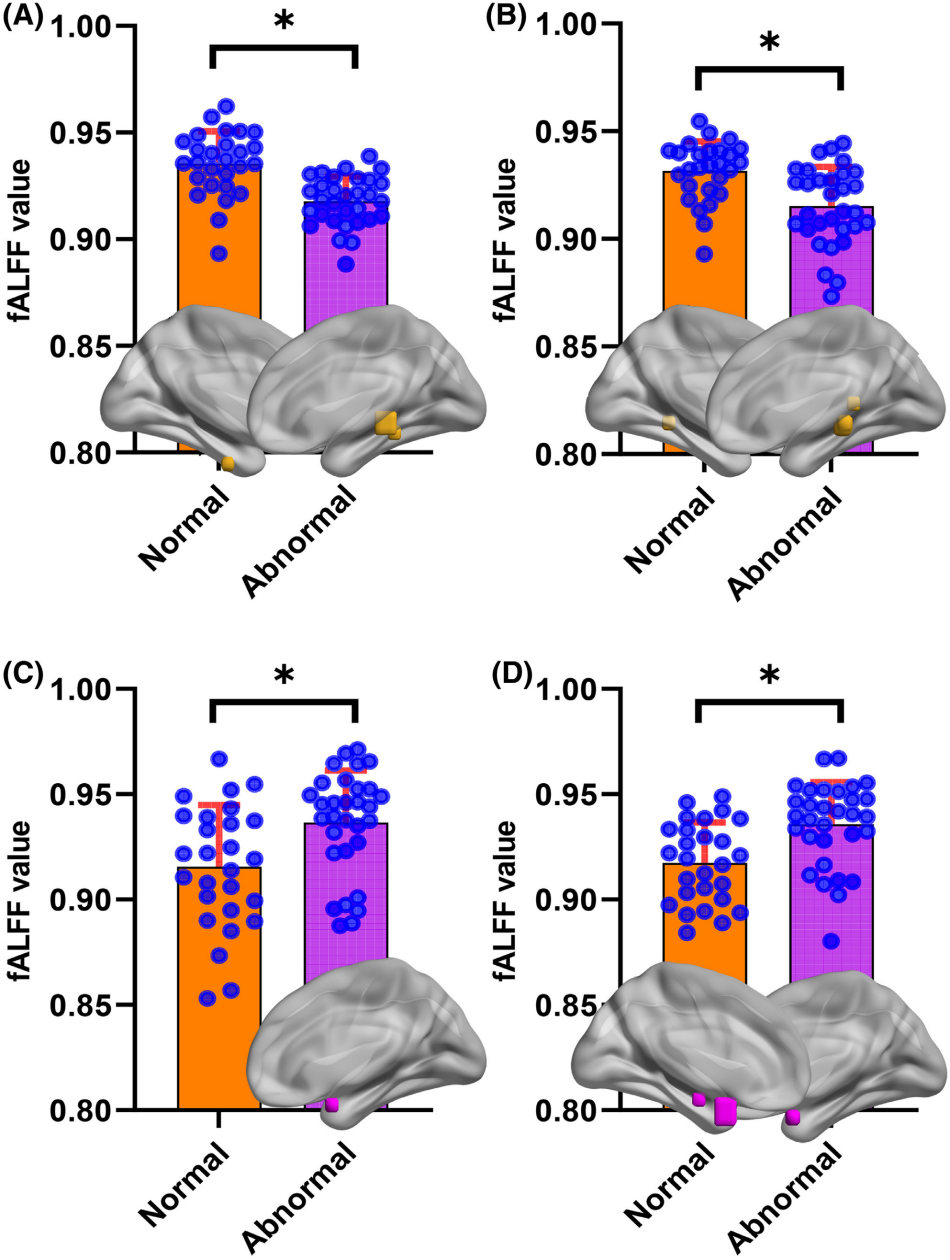
Quantitative results of fALFF values reveal differences in spontaneous brain activity in the hippocampal region between the cognitively abnormal and the NC groups (*p < 0.05; fALFF, fractional amplitude of low‐frequency fluctuation). (A) The fALFF‐reduced brain regions in the hippocampus of patients with cognitively abnormal (uncorrected for confounders). (B) The fALFF‐reduced brain regions (corrected for confounders). (C) The fALFF‐enhanced brain regions uncorrected for confounders. (D) The fALFF‐enhanced distribution of the brain regions corrected for confounders.

The study found that the cognitive level of PD patients could be effectively characterized by their spontaneous brain activity in the hippocampus region. To reveal the baseline level of spontaneous brain activity in the hippocampus of the PD patients with NC, we further compared the differences in fALFF in the hippocampus region between NC and cognitively abnormal patients and quantified the fALFF values. Specifically, Figure 4 presents the fALFF‐reduced (Figure 4A,B) and ‐enhanced (Figure 4C,D) brain regions of patients with cognitively abnormal, both uncorrected (Figure 4A,C) and corrected (Figure 4B,D) for confounders. The fALFF quantification showed that compared to the cognitively abnormal group, the NC group was 0.936 ± 0.015 vs. 0.918 ± 0.012 (Figure 4A) and 0.916 ± 0.029 vs. 0.937 ± 0.025 (Figure 4C), while corrected for confounders was 0.932 ± 0.014 vs. 0.915 ± 0.018 (Figure 4B) and 0.918 ± 0.019 vs. 0.936 ± 0.020 (Figure 4D).

### 3.6 Making ROIs in different brain regions

**FIGURE 5:**
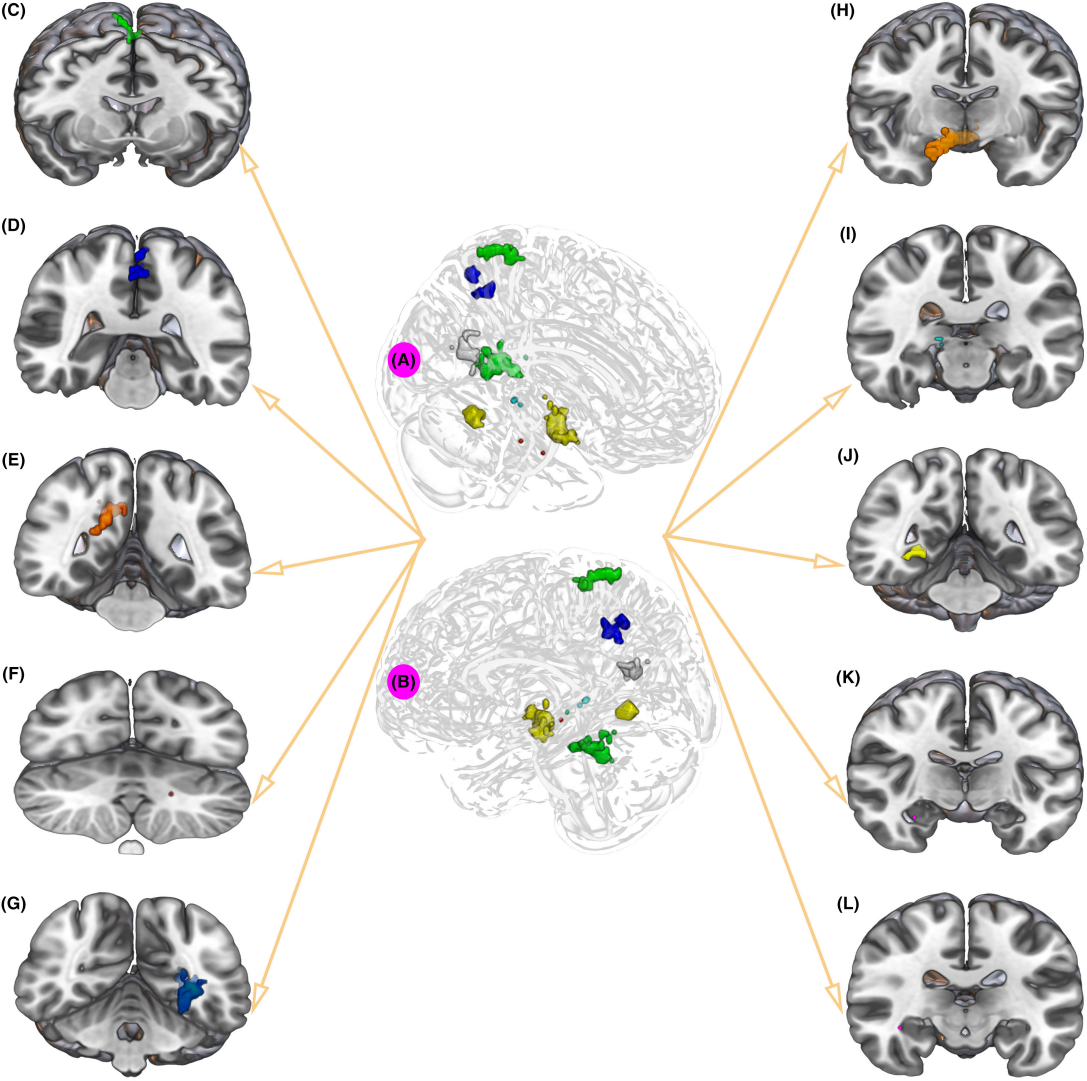
Defining the ROIs. (A) Right‐side view of 10 ROIs. (B) Left side view of 10 ROIs. (C) Paracentral lobule, Postcentral Gyrus (ROI 1). (D) Precuneus (ROI 2). (E) Calcarine, Posterior Cingulate (ROI 3). (F) Left Hippocampus (ROI 4). (G) Parahippocampal Gyrus (ROI 5). (H) Midbrain, Hippocampus, Parahippocampal Gyrus (ROI 6). (I) Midbrain, Right Hippocampus (ROI 7). (J) Parahippocampal Gyrus, Fusiform Gyrus, Gyrus Lingualis (ROI 8). (K) Hippocampus, Parahippocampal Gyrus (ROI 9). (L) Right Hippocampus, Temporal Lobe (ROI 10).

We overlay the differential brain regions expressed by Figures 1, 2, 3 and obtained 10 ROIs in Figure 5 according to the spatial distribution, including Paracentral lobule, Postcentral Gyrus (ROI 1), Precuneus (ROI 2), Calcarine, Posterior Cingulate (ROI 3), Left Hippocampus (ROI 4), Parahippocampa Gyrus (ROI 5), Midbrain, Hippocampus, Parahippocampa Gyrus (ROI 6), Midbrain, Right Hippocampus (ROI 7), Parahippocampa Gyrus, Fusiform Gyrus, Gyrus Lingualis (ROI 8), Hippocampus, Parahippocampa Gyrus (ROI 9), Right Hippocampus, Temporal Lobe (ROI 10) (Figure 5, Table 3).

**TABLE 3:** The name of the brain regions corresponding to the ROIs.

Regions of interest	Peak MNI	Name
	*x*/*y*/*z*	
ROI 1	1/−35/70	Paracentral Lobule, Postcentral Gyrus
ROI 2	1/−50/48	Precuneus
ROI 3	21/−50/14	Calcarine, Posterior Cingulate
ROI 4	−18/−36/6	Left Hippocampus
ROI 5	−30/−56/−5	Parahippocampa Gyrus
ROI 6	8/−8/−14	Midbrain, Hippocampus, Parahippocampa Gyrus
ROI 7	15/−28/−5	Midbrain, Right Hippocampus
ROI 8	27/−47/−8	Parahippocampa Gyrus, Fusiform Gyrus, Gyrus Lingualis
ROI 9	30/−9/−24	Hippocampus, Parahippocampa Gyrus
ROI 10	39/−18/−15	Right Hippocampus, Temporal Lobe

Abbreviation: MNI, Montreal Neurological Institute.

### 3.7 Comparison of fALFF of 10 ROIs between different cognitive groups

**FIGURE 6:**
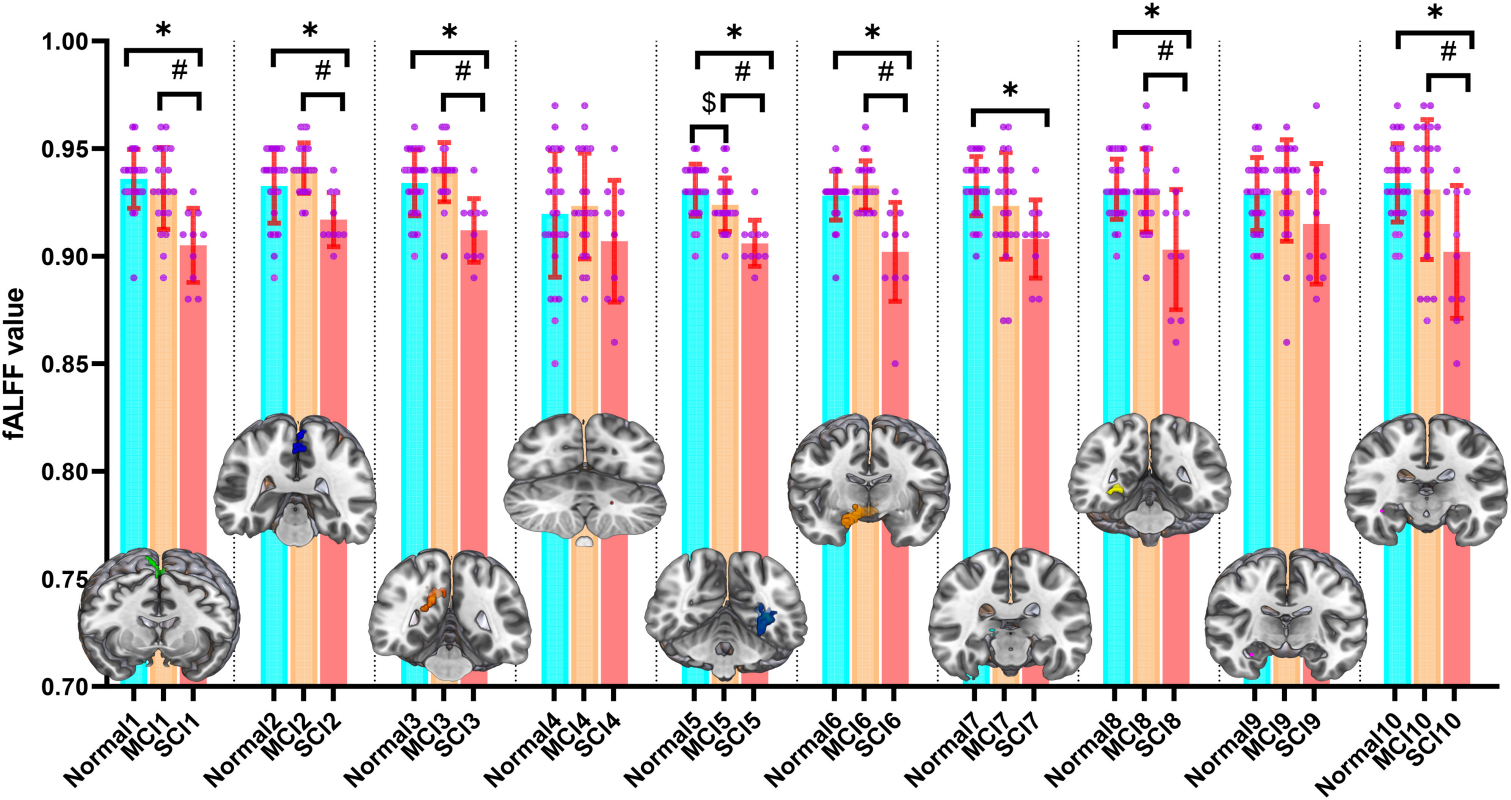
Comparison of fALFF of 10 ROIs between different cognitive groups; The order of *X*‐axis numbers corresponds to the order of ROIs (#*$*p* < 0.05; fALFF, fractional amplitude of low‐frequency fluctuation; MCI, mild cognitive impairment; SCI, severe cognitive impairment).

The fALFF of three cognitive groups were collected according to the 10 ROIs in Figure 6, and the differences between groups were compared (Figure 6). When comparing PD patients with MCI and NC, there were differences in fALFF only including ROI 5. When comparing PD patients with SCI and NC, there were differences in fALFF of 8 regions including ROI 1, ROI 2, ROI 3, ROI 5, ROI 6, ROI 7, ROI 8 and ROI 10. When comparing PD patients with SCI and MCI, there were differences in fALFF of 7 regions including ROI 1, ROI 2, ROI 3, ROI 5, ROI 6, ROI 8, and ROI 10. ROI 5 was the only region with statistically significant differences in fALFF among the three cognitive groups. We found that ROI 5 had the highest fALFF in PD patients with NC and the lowest fALFF in patients with SCI.

### 3.8 The potential ability to identify cognitive impairment by ROIs

**FIGURE 7:**
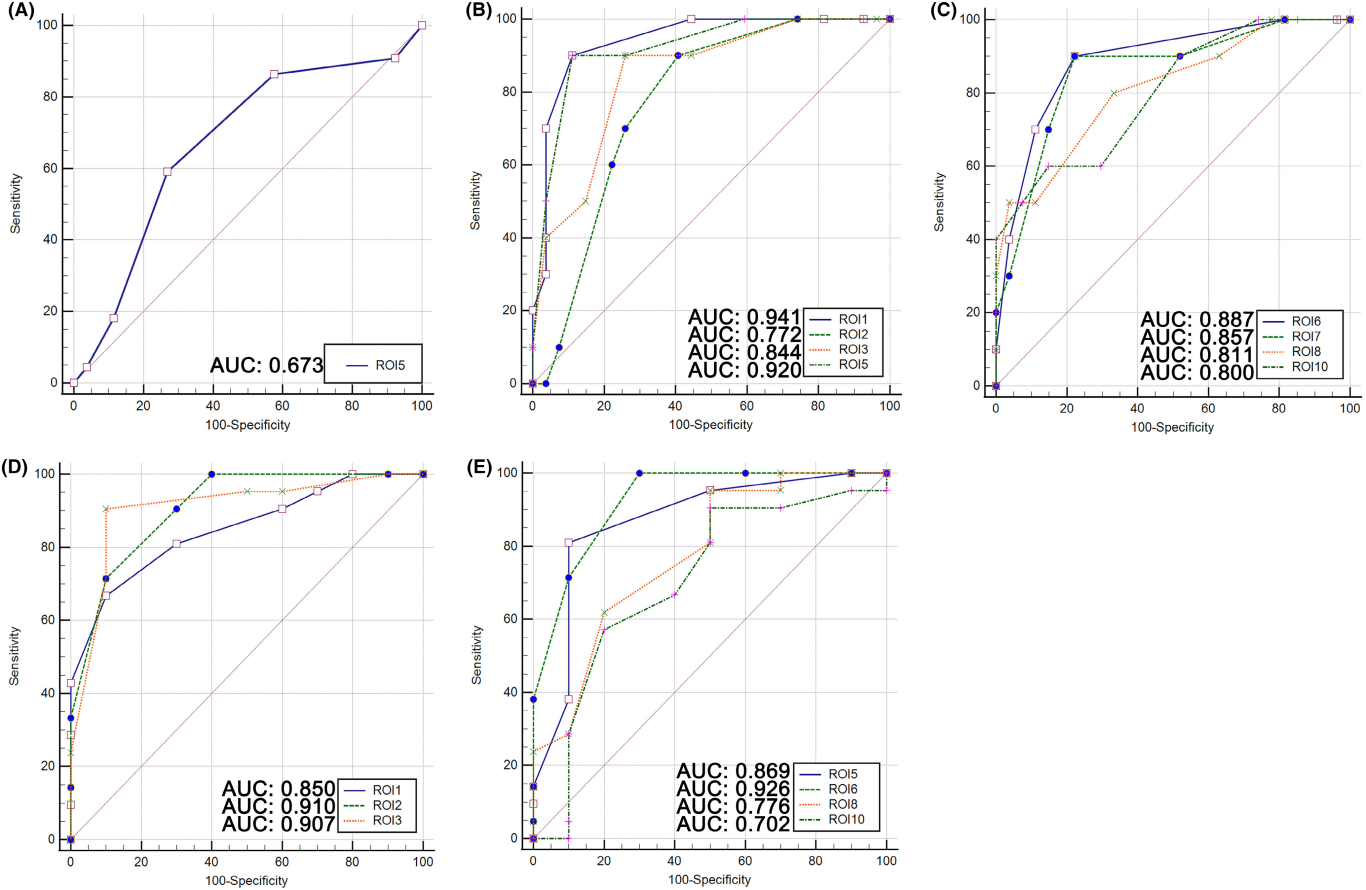
The potential ability of ROIs to identify between cognitive impairments (AUC: area under the curve). (A) Distinguishing patients with MCI from NC by ROI 5. (B, C) distinguishing patients with SCI from NC by ROI 1, ROI 2, ROI 3, ROI 5, ROI 6, ROI 7, ROI 8, ROI 10. (D, E) distinguishing patients with SCI from MCI by ROI 1, ROI 2, ROI 3, ROI 5, ROI 6, ROI 8, ROI 10.

We produce ROC‐AUC for the evaluation of the potential of ROIs to identify cognitive impairment. The AUC of fALFF of ROI 5 in distinguishing PD patients with MCI from NC was 0.673 (Figure 7A). The AUC of fALFF of ROI 1, ROI 2, ROI 3, ROI 5, ROI 6, ROI 7, ROI 8, ROI 10 in distinguishing PD patients with SCI from NC was 0.941, 0.772, 0.844, 0.920, 0.887, 0.857, 0.811, 0.800, respectively (Figure 7B,C). The AUC of fALFF of ROI 1, ROI 2, ROI 3, ROI 5, ROI 6, ROI 8, ROI 10 in distinguishing PD patients with SCI from MCI was 0.850, 0.910, 0.907, 0.869, 0.926, 0.776, 0.702, respectively (Figure 7D,E). The results revealed that parahippocampal gyrus (ROI 5) was the only region that identified PD patients with MCI and NC. The paracentral lobule, postcentral gyrus (ROI 1) was the best region that identified PD patients with SCI and NC, with an AUC of 0.941. The midbrain, hippocampus, and parahippocampa gyrus (ROI 6) was the best region that identified PD patients with SCI and MCI, with an AUC of 0.926.

## 4 DISCUSSION

### 4.1 fALFF is an effective method to assess brain activity in PD patients

The risk of dementia in PD patients is 5–6 times higher than that in normal peers.^11^, ^26^, ^27^, ^28^ With the progress of the disease, most PD patients will eventually be diagnosed with dementia. The prevalence of dementia in PD patients was approximately 30%, with an incidence rate of 24.3/1000/year (95% confidence interval, 7.7–58).^6^ Cognitive impairment was a manifestation in the early stage of dementia. Early detection and intervention of cognitive impairment could improve the quality of life of PD patients.^29^, ^30^ The research on brain activity associated with cognitive impairment of PD patients had important implications not only for the treatment and management of PD patients but also for further understanding the pathophysiological mechanisms of PD. We used RS‐fMRI technology to collect brain activity parameters and applied network perspective to study the differences in brain activity in PD patients and find the early warning methods of PD‐MCI diagnosis.

The resting state was a state in which the brain was quiet, relaxed, and awake without performing cognitive tasks, and was the most basic and essential state of various complex states of the brain. The amplitude of low‐frequency fluctuation (ALFF) was a signal intensity of the blood oxygen level dependence (BOLD) sequence, which was used to describe the brain activity intensity of a single voxel and was an important feature in describing resting‐state images.^31^ In order to reduce the sensitivity of ALFF to physiological noise and improve the sensitivity and specificity of detecting spontaneous brain activity, the fractional amplitude of low‐frequency fluctuations (fALFF) was used in our research.

From the perspective of brain activity, the use of ALFF to study non‐motor characteristics of PD patients was the hot research. In the study of anxiety characteristics in PD, Zhang study found that the fALFF of the left cerebellum, cerebellum posterior lobe, bilateral temporal cortex, and brainstem were higher, while the fALFF of bilateral inferior gyrus, bilateral basal ganglia areas, and left inferior parietal lobule were lower. Right, precuneus, and left caudate were correlated with the Hamilton Anxiety Scale.^32^ By monitoring different brain regions ALFF, Criaud found that anxiety in PD was associated with the over‐activation of the amygdala and impaired inter‐relationship of regions involved in behavior (i.e. medial prefrontal cortex, insula) and motor control (i.e. basal ganglia).^33^ The fALFF of bilateral putamen in PD patients with cognitive impairment was low, and the fALFF of left putamen was negatively correlated with the scores of PD‐MCI patients on the Movement Disorder Society‐Unified Parkinson Disease Rating Scale Part III.^34^ Monitoring ALFF in the bilateral primary motor cortex, the occipital cortex, the cerebellum, and the basal ganglia could predict the effect of dopaminergic therapy in PD patients.^35^

### 4.2 The hippocampus is the important brain region for cognitive impairment in PD

We analyzed the correlation between whole brain fALFF by voxels and cognition in PD patients. We found similar results in brain regions to previous studies, including the brainstem, hippocampal structure, limbic lobe, amygdala, occipital lobe, etc.^36^, ^37^ These results indicated that the above brain regions were closely related to cognition, attention, and memory.^38^

About 26.7% (18.9%–38.2%) of non‐demented PD patients were considered MCI,^39^ and an even higher proportion of 40% was considered MCI.^40^ In order to find the characteristic brain regions of PD‐MCI, we compared fALFF between PD patients with MCI and NC. Unfortunately, there was no statistically significant difference in fALFF between the two groups regardless of adjustment for confounding factors. A previous study demonstrated that compared with PD‐NC, PD‐MCI showed significantly increased ALFF in the right inferior frontal gyrus and left fusiform gyrus.^31^

Results found that hippocampal structure was not only a brain region related to cognition in PD patients but also a differential fALFF brain region in PD‐SCI. The hippocampus was closely related to learning, memory, emotional response, and other brain neural activities, and it was also an important target for research on cognitive impairment in PD patients because the temporal lobe has been identified as crucial for encoding and memory consolidation.^41^, ^42^

In terms of magnetic resonance imaging, previous studies have demonstrated an association between hippocampal atrophy and MCI, as well as dementia.^43^, ^44^, ^45^ We innovatively analyzed the correlation between hippocampus fALFF by voxels and cognition in PD patients. It was found that regardless of confounding factors such as age, gender, handedness, education level, the hippocampal region was a stable representative brain region of cognition.

### 4.3 Feasibility of parahippocampal gyrus in predicting cognitive impairment in PD patients

The purpose and methods of the current research were limited to speculating the effect of hippocampal atrophy on cognitive impairment in PD patients from the perspective of imaging. Gray matter atrophy of the hippocampus in PD patients resulted in impairment of functional connectivity, associated with different stages of cognitive impairment.^46^, ^47^ At present, some researchers explored the function of the hippocampus in PD patients from the perspective of brain activity. In the study of non‐motor symptoms of PD patients, Xu found an abnormal degree of centrality value in the hippocampus, and parahippocampus was observed separately in the conventional band and in the slow‐4 band in PD with apathy, which confirmed the density of internal functional connections in hippocampal decreased.^48^ Zi performed statistical differences in fALFF between patients in PD with excessive daytime sleepiness and analyzed the changes in the functional connection within the whole brain. It was found that the functional connectivity in the right hippocampus/parahippocampal was decreased, which resulted in damage to the dopaminergic circuits in the limbic system and subsequently inhibited the arousal mechanism.^49^ However, Wang found that ALFF was elevated in the hippocampal structure in PD patients, and the hippocampus was an important structure for studying PD.^50^

By comparing the fALFF of three cognitive groups, we found that the parahippocampal gyrus corresponding to the ROI 5 brain region was the different brain region of the three cognitive groups, and it was also the only brain region to identify MCI from NC. The parahippocampal gyrus was an important and active region of the limbic system, whose main functions included creating memories and recalling visual scenes. The research found that impaired function of the parahippocampal gyrus in PD‐MCI led to memory impairment^31^ and showed that parahippocampal gyrus was essential for discovering connections between things and finding target objects.^51^

We also found that the fALFF of parahippocampal gyrus of PD patients with SCI decreased more significantly than that of PD patients with MCI and NC. Previous evidence of hippocampal atrophy combined with our results of decreased parahippocampal gyrus activity might account for and predict the cognitive impairment in PD patients. Similarly, in the study of vascular dementia and Alzheimer's disease, it was found that abnormal brain activities in different parts of brain were related to pathological processes, which provided theoretical support for explaining the mechanism of cognitive impairment in imaging perspective.^52^, ^53^ The study found that sharp ripples are a manifestation of interactions between the hippocampus and cortical loops, which are closely linked to cognition and memory.^54^ However, whether similar cognitive loop abnormalities exist in the PD co‐morbid cognitively impaired population warrants elucidation in terms of functional versus causal connectivity.

In order to explore the ability of brain activity in the parahippocampal gyrus to identify cognitive impairment, we found that the fALFF of parahippocampal gyrus of PD patients with MCI decreased more significantly than that of PD patients with SCI, which indicated that the brain activity of parahippocampal gyrus decreased, and its functional impairment was closely related to cognitive impairment. The fALFF of parahippocampal gyrus was 0.673 of AUC for identifying MCI and NC, 0.920 of AUC for identifying SCI and NC, and 0.869 of AUC for identifying SCI and MCI. From our perspective, the ability of fALFF of parahippocampal gyrus in identifying SCI from NC and MCI was acceptable, but the AUC for identifying the most important MCI from NC was 0.673, and its ability to identify was ordinary. This may be a potential biomarker worth combining with other indicators.

### 4.4 Limitations need to be discussed

Firstly, common and typical results of brain activity in PD patients were found based on the current sample size, but the robustness of the results was worth further verifying in a larger sample size. Secondly, brain activity in the parahippocampal gyrus was found to be an effective region for cognitive stratification in PD patients, but the brain regions (functional connectivity) affected by the abnormal parahippocampal gyrus activity were still unclear. It was suggested that regional homogeneity and functional connectivity methods were recommended to explore the relationship between brain regions in future studies. In addition, it is a consensus that structure determines function. From the perspective of brain activity, we showed the new discoveries, however, it was beneficial to further understand the pathological mechanism of PD patients evolving into different cognitive states by multimodal methods finding the changes between structure and function. Finally, abnormal brain activity in the parahippocampal gyrus could early identify PD‐MCI and became an early warning indicator, however, its ability to identify was ordinary. We suggested that in further research, it was necessary to combine other biochemical indicators, cerebral blood flow, and other parameters to comprehensively analyze.

## 5 CONCLUSIONS

According to the research, the cognitive impairment of PD patients was associated with age and gender. By comparing the fALFF of three cognitive groups, discovery of the parahippocampal gyrus as an anchored differential brain region and it was also the only brain region to identify MCI from NC. It was revealed parahippocampal gyrus was the prospective brain region for recognizing cognitive impairment in PD, specifically for identifying MCI. In view of the ordinary ability of fALFF of parahippocampal gyrus to identify the most important MCI from NC, it was necessary to combine fALFF of parahippocampal gyrus with other indicators for early warning study.

## FUNDING INFORMATION

The funding was provided by the Fundamental Research Funds for the Central Universities (2022CDJYGRH‐015); the National Natural Science Foundation of Chongqing (CSTB2022NSCQ‐MSX1503); Medical Research Project of Science and Technology Bureau and Health Commission (2023MSXM076); and Project of Chongqing Key Laboratory of Emergency Medicine (2024RCCX01).

## CONFLICT OF INTEREST STATEMENT

The authors report no conflicts of interest in this work.

## Supporting information


Table S1

Table S2

Table S3

Table S4

Table S5

Table S6

Table S7

Table S8

## Data Availability

The data that support the findings could be available from the corresponding author.

## References

[1] Pfeiffer RF . Non‐motor symptoms in Parkinson's disease. Parkinsonism Relat Disord. 2016;22 Suppl 1:S119‐S122. doi:10.1016/j.parkreldis.2015.09.004 26372623

[2] Liu WM , Lin RJ , Yu RL , et al. The impact of nonmotor symptoms on quality of life in patients with Parkinson's disease in Taiwan. Neuropsychiatr Dis Treat. 2015;11:2865‐2873. doi:10.2147/NDT.S88968 26635475 PMC4646598

[3] Fan Y , Liang X , Han L , et al. Determinants of quality of life according to cognitive status in Parkinson's disease. Front Aging Neurosci. 2020;12:269. doi:10.3389/fnagi.2020.00269 32973491 PMC7468499

[4] Bugalho P , Ladeira F , Barbosa R , et al. Motor and non‐motor function predictors of mortality in Parkinson's disease. J Neural Transm (Vienna). 2019;126(11):1409‐1415. doi:10.1007/s00702-019-02055-3 31385098

[5] Petersen RC . Mild cognitive impairment as a diagnostic entity. J Intern Med. 2004;256(3):183‐194. doi:10.1111/j.1365-2796.2004.01388.x 15324362

[6] Nicoletti A , Luca A , Baschi R , et al. Incidence of mild cognitive impairment and dementia in Parkinson's disease: the Parkinson's disease cognitive impairment study. Front Aging Neurosci. 2019;11:21. doi:10.3389/fnagi.2019.00021 30800065 PMC6376919

[7] Litvan I , Goldman JG , Troster AI , et al. Diagnostic criteria for mild cognitive impairment in Parkinson's disease: Movement Disorder Society task force guidelines. Mov Disord. 2012;27(3):349‐356. doi:10.1002/mds.24893 22275317 PMC3641655

[8] Geurtsen GJ , Hoogland J , Goldman JG , et al. Parkinson's disease mild cognitive impairment: application and validation of the criteria. J Parkinsons Dis. 2014;4(2):131‐137. doi:10.3233/JPD-130304 24296865 PMC4380013

[9] Goldman JG , Holden SK , Litvan I , McKeith I , Stebbins GT , Taylor JP . Evolution of diagnostic criteria and assessments for Parkinson's disease mild cognitive impairment. Mov Disord. 2018;33(4):503‐510. doi:10.1002/mds.27323 29488270 PMC12570294

[10] Cholerton B , Johnson CO , Fish B , et al. Sex differences in progression to mild cognitive impairment and dementia in Parkinson's disease. Parkinsonism Relat Disord. 2018;50:29‐36. doi:10.1016/j.parkreldis.2018.02.007 29478836 PMC5943177

[11] Williams‐Gray CH , Evans JR , Goris A , et al. The distinct cognitive syndromes of Parkinson's disease: 5 year follow‐up of the CamPaIGN cohort. Brain. 2009;132(Pt 11):2958‐2969. doi:10.1093/brain/awp245 19812213

[12] Foltynie T , Goldberg TE , Lewis SG , et al. Planning ability in Parkinson's disease is influenced by the COMT val158met polymorphism. Mov Disord. 2004;19(8):885‐891. doi:10.1002/mds.20118 15300652

[13] Tropea TF , Xie SX , Rick J , et al. APOE, thought disorder, and SPARE‐AD predict cognitive decline in established Parkinson's disease. Mov Disord. 2018;33(2):289‐297. doi:10.1002/mds.27204 29168904 PMC5809205

[14] Xu Y , Yang J , Hu X , Shang H . Voxel‐based meta‐analysis of gray matter volume reductions associated with cognitive impairment in Parkinson's disease. J Neurol. 2016;263(6):1178‐1187. doi:10.1007/s00415-016-8122-3 27113603

[15] Mihaescu AS , Masellis M , Graff‐Guerrero A , et al. Brain degeneration in Parkinson's disease patients with cognitive decline: a coordinate‐based meta‐analysis. Brain Imaging Behav. 2019;13(4):1021‐1034. doi:10.1007/s11682-018-9922-0 29971686

[16] Arslan DB , Gurvit H , Genc O , et al. The cerebral blood flow deficits in Parkinson's disease with mild cognitive impairment using arterial spin labeling MRI. J Neural Transm (Vienna). 2020;127(9):1285‐1294. doi:10.1007/s00702-020-02227-6 32632889

[17] Azamat S , Betul Arslan D , Erdogdu E , et al. Detection of visual and frontoparietal network perfusion deficits in Parkinson's disease dementia. Eur J Radiol. 2021;144:109985. doi:10.1016/j.ejrad.2021.109985 34619619

[18] Hughes D , Judge C , Murphy R , et al. Association of blood pressure lowering with incident dementia or cognitive impairment: a systematic review and meta‐analysis. JAMA. 2020;323(19):1934‐1944. doi:10.1001/jama.2020.4249 32427305 PMC7237983

[19] Suzuki Y , Tsubaki T , Nakaya K , et al. New balance capability index as a screening tool for mild cognitive impairment. BMC Geriatr. 2023;23(1):74. doi:10.1186/s12877-023-03777-6 36739383 PMC9899403

[20] Dove E , Astell AJ . Kinect project: people with dementia or mild cognitive impairment learning to play group motion‐based games. Alzheimers Dement (N Y). 2019;5:475‐482. doi:10.1016/j.trci.2019.07.008 31650003 PMC6804497

[21] Nasreddine ZS , Phillips NA , Bedirian V , et al. The montreal cognitive assessment, MoCA: a brief screening tool for mild cognitive impairment. J Am Geriatr Soc. 2005;53(4):695‐699. doi:10.1111/j.1532-5415.2005.53221.x 15817019

[22] Dawson BK , Fereshtehnejad SM , Anang JBM , et al. Office‐based screening for dementia in Parkinson disease: the montreal Parkinson risk of dementia scale in 4 longitudinal cohorts. JAMA Neurol. 2018;75(6):704‐710. doi:10.1001/jamaneurol.2018.0254 29582054 PMC5885166

[23] Zhang J , Zhang E , Yuan C , et al. Abnormal default mode network could be a potential prognostic marker in patients with disorders of consciousness. Clin Neurol Neurosurg. 2022;218:107294. doi:10.1016/j.clineuro.2022.107294 35597165

[24] Zang YF , He Y , Zhu CZ , et al. Altered baseline brain activity in children with ADHD revealed by resting‐state functional MRI. Brain and Development. 2007;29(2):83‐91. doi:10.1016/j.braindev.2006.07.002 16919409

[25] Zou QH , Zhu CZ , Yang Y , et al. An improved approach to detection of amplitude of low‐frequency fluctuation (ALFF) for resting‐state fMRI: fractional ALFF. J Neurosci Methods. 2008;172(1):137‐141. doi:10.1016/j.jneumeth.2008.04.012 18501969 PMC3902859

[26] Williams‐Gray CH , Mason SL , Evans JR , et al. The CamPaIGN study of Parkinson's disease: 10‐year outlook in an incident population‐based cohort. J Neurol Neurosurg Psychiatry. 2013;84(11):1258‐1264. doi:10.1136/jnnp-2013-305277 23781007

[27] Buter TC , van den Hout A , Matthews FE , Larsen JP , Brayne C , Aarsland D . Dementia and survival in Parkinson disease: a 12‐year population study. Neurology. 2008;70(13):1017‐1022. doi:10.1212/01.wnl.0000306632.43729.24 18362281

[28] Hely MA , Reid WG , Adena MA , et al. The Sydney multicenter study of Parkinson's disease: the inevitability of dementia at 20 years. Mov Disord. 2008;23(6):837‐844. doi:10.1002/mds.21956 18307261

[29] Aarsland D , Kurz MW . The epidemiology of dementia associated with Parkinson disease. J Neurol Sci. 2010;289(1–2):18‐22. doi:10.1016/j.jns.2009.08.034 19733364

[30] Leroi I , McDonald K , Pantula H , Harbishettar V . Cognitive impairment in Parkinson disease: impact on quality of life, disability, and caregiver burden. J Geriatr Psychiatry Neurol. 2012;25(4):208‐214. doi:10.1177/0891988712464823 23172765

[31] Wang Z , Jia X , Chen H , Feng T , Wang H . Abnormal spontaneous brain activity in early Parkinson's disease with mild cognitive impairment: a resting‐state fMRI study. Front Physiol. 2018;9:1093. doi:10.3389/fphys.2018.01093 30154730 PMC6102476

[32] Zhang P , Gao Y , Hu Y , et al. Altered fractional amplitude of low‐frequency fluctuation in anxious Parkinson's disease. Brain Sci. 2023;13(1):87. doi:10.3390/brainsci13010087 36672068 PMC9857220

[33] Criaud M , Kim JH , Zurowski M , et al. Anxiety in Parkinson's disease: abnormal resting activity and connectivity. Brain Res. 2021;1753:147235. doi:10.1016/j.brainres.2020.147235 33412150

[34] Rong S , Zhang P , He C , et al. Abnormal neural activity in different frequency bands in Parkinson's disease with mild cognitive impairment. Front Aging Neurosci. 2021;13:709998. doi:10.3389/fnagi.2021.709998 34489679 PMC8417797

[35] Yang B , Wang X , Mo J , et al. The amplitude of low‐frequency fluctuation predicts levodopa treatment response in patients with Parkinson's disease. Parkinsonism Relat Disord. 2021;92:26‐32. doi:10.1016/j.parkreldis.2021.10.003 34666272

[36] Chen HM , Wang ZJ , Fang JP , et al. Different patterns of spontaneous brain activity between tremor‐dominant and postural instability/gait difficulty subtypes of Parkinson's disease: a resting‐state fMRI study. CNS Neurosci Ther. 2015;21(10):855‐866. doi:10.1111/cns.12464 26387576 PMC6493074

[37] Pan P , Zhang Y , Liu Y , Zhang H , Guan DN , Xu Y . Abnormalities of regional brain function in Parkinson's disease: a meta‐analysis of resting state functional magnetic resonance imaging studies. Sci Rep. 2017;7:40469. doi:10.1038/srep40469 28079169 PMC5228032

[38] Hou Y , Yang J , Luo C , et al. Dysfunction of the default mode network in drug‐naive Parkinson's disease with mild cognitive impairments: a resting‐state fMRI study. Front Aging Neurosci. 2016;8:247. doi:10.3389/fnagi.2016.00247 27833548 PMC5080293

[39] Litvan I , Aarsland D , Adler CH , et al. MDS task force on mild cognitive impairment in Parkinson's disease: critical review of PD‐MCI. Mov Disord. 2011;26(10):1814‐1824. doi:10.1002/mds.23823 21661055 PMC3181006

[40] Baiano C , Barone P , Trojano L , Santangelo G . Prevalence and clinical aspects of mild cognitive impairment in Parkinson's disease: a meta‐analysis. Mov Disord. 2020;35(1):45‐54. doi:10.1002/mds.27902 31743500

[41] Villar‐Conde S , Astillero‐Lopez V , Gonzalez‐Rodriguez M , et al. The human hippocampus in Parkinson's disease: an integrative stereological and proteomic study. J Parkinsons Dis. 2021;11(3):1345‐1365. doi:10.3233/JPD-202465 34092653 PMC8461741

[42] Squire LR , Stark CE , Clark RE . The medial temporal lobe. Annu Rev Neurosci. 2004;27:279‐306. doi:10.1146/annurev.neuro.27.070203.144130 15217334

[43] Camicioli R , Moore MM , Kinney A , Corbridge E , Glassberg K , Kaye JA . Parkinson's disease is associated with hippocampal atrophy. Mov Disord. 2003;18(7):784‐790. doi:10.1002/mds.10444 12815657

[44] Chung SJ , Shin JH , Cho KH , et al. Subcortical shape analysis of progressive mild cognitive impairment in Parkinson's disease. Mov Disord. 2017;32(10):1447‐1456. doi:10.1002/mds.27106 28737237

[45] Melzer TR , Watts R , MacAskill MR , et al. Grey matter atrophy in cognitively impaired Parkinson's disease. J Neurol Neurosurg Psychiatry. 2012;83(2):188‐194. doi:10.1136/jnnp-2011-300828 21890574

[46] Kandiah N , Zainal NH , Narasimhalu K , et al. Hippocampal volume and white matter disease in the prediction of dementia in Parkinson's disease. Parkinsonism Relat Disord. 2014;20(11):1203‐1208. doi:10.1016/j.parkreldis.2014.08.024 25258331

[47] Chen FX , Kang DZ , Chen FY , et al. Gray matter atrophy associated with mild cognitive impairment in Parkinson's disease. Neurosci Lett. 2016;617:160‐165. doi:10.1016/j.neulet.2015.12.055 26742642

[48] Xu H , Zhang M , Wang Z , Yang Y , Chang Y , Liu L . Abnormal brain activities in multiple frequency bands in Parkinson's disease with apathy. Front Neurosci. 2022;16:975189. doi:10.3389/fnins.2022.975189 36300172 PMC9589053

[49] Zi Y , Cai S , Tan C , et al. Abnormalities in the fractional amplitude of low‐frequency fluctuation and functional connectivity in Parkinson's disease with excessive daytime sleepiness. Front Aging Neurosci. 2022;14:826175. doi:10.3389/fnagi.2022.826175 35865749 PMC9294344

[50] Wang Z , Liu Y , Ruan X , et al. Aberrant amplitude of low‐frequency fluctuations in different frequency bands in patients with Parkinson's disease. Front Aging Neurosci. 2020;12:576682. doi:10.3389/fnagi.2020.576682 33343329 PMC7744880

[51] Owen AM , Milner B , Petrides M , Evans AC . A specific role for the right parahippocampal gyrus in the retrieval of object‐location: a positron emission tomography study. J Cogn Neurosci. 1996;8(6):588‐602. doi:10.1162/jocn.1996.8.6.588 23961986

[52] Fu Y , Luo X , Zeng Q , et al. Effects of anosognosia on static and dynamic amplitudes of low‐frequency fluctuation in mild cognitive impairment. Front Aging Neurosci. 2021;13:705097. doi:10.3389/fnagi.2021.705097 35221980 PMC8867082

[53] Li H , Jia X , Li Y , Jia X , Yang Q . Aberrant amplitude of low‐frequency fluctuation and degree centrality within the default mode network in patients with vascular mild cognitive impairment. Brain Sci. 2021;11(11):1534. doi:10.3390/brainsci11111534 34827533 PMC8615791

[54] Ramirez‐Villegas JF , Besserve M , Murayama Y , Evrard HC , Oeltermann A , Logothetis NK . Coupling of hippocampal theta and ripples with pontogeniculooccipital waves. Nature. 2021;589(7840):96‐102. doi:10.1038/s41586-020-2914-4 33208951

