# Supplementary material for: Spontaneous brain activity in the hippocampal regions could characterize cognitive impairment in patients with Parkinson's disease
Source: CNS Neurosci Ther. 2024 Apr 7;30(4):e14706. doi: 10.1111/cns.14706 (PMC10999557; doi:10.1111/cns.14706)
Supplement: Supplementary file 1 — Table S1 [file CNS-30-e14706-s007.doc]

**Table S1**. Unadjusted for confounders, differences in brain regions among the NC, MCI, and SCI groups.

This report is based on CUI Xu's xjview. (http://www.alivelearn.net/xjview/)

Revised by YAN Chao-Gan and ZHU Wei-Xuan 20091108: suitable for different Cluster Connectivity Criterion: surface connected, edge connected, corner connected.

Number of clusters found: 1

----------------------

Cluster 1

Number of voxels: 48

Peak MNI coordinate: 9 -9 -15

Peak MNI coordinate region: // Right Brainstem // Midbrain // undefined // undefined // undefined // undefined

Peak intensity: 13.7355

# voxels structure

48 --TOTAL # VOXELS--

18 Right Cerebrum

16 Midbrain

15 Limbic Lobe

15 Parahippocampa Gyrus

11 Gray Matter

9 Right Brainstem

8 brodmann area 34

7 ParaHippocampal_R (aal)

7 Amygdala_R (aal)

7 Left Brainstem

3 Amygdala

1 Frontal Lobe

1 Subcallosal Gyrus

>>
