# Supplementary material for: Spontaneous brain activity in the hippocampal regions could characterize cognitive impairment in patients with Parkinson's disease
Source: CNS Neurosci Ther. 2024 Apr 7;30(4):e14706. doi: 10.1111/cns.14706 (PMC10999557; doi:10.1111/cns.14706)
Supplement: Supplementary file 2 — Table S2 [file CNS-30-e14706-s001.doc]

**Table S2**. Adjusted for confounders, differences in brain regions among the NC, MCI, and SCI groups.

This report is based on CUI Xu's xjview. (http://www.alivelearn.net/xjview/)

Revised by YAN Chao-Gan and ZHU Wei-Xuan 20091108: suitable for different Cluster Connectivity Criterion: surface connected, edge connected, corner connected.

Number of clusters found: 1

----------------------

Cluster 1

Number of voxels: 41

Peak MNI coordinate: 12 -6 -21

Peak MNI coordinate region: // Right Cerebrum // undefined // undefined // undefined // undefined // undefined

Peak intensity: 14.6006

# voxels structure

41 --TOTAL # VOXELS--

16 Midbrain

13 Right Cerebrum

10 Right Brainstem

9 Parahippocampa Gyrus

9 Limbic Lobe

7 Gray Matter

6 Amygdala_R (aal)

6 brodmann area 34

6 Left Brainstem

4 ParaHippocampal_R (aal)

2 Frontal Lobe

2 Subcallosal Gyrus

1 Amygdala

>>
