# Supplementary material for: Spontaneous brain activity in the hippocampal regions could characterize cognitive impairment in patients with Parkinson's disease
Source: CNS Neurosci Ther. 2024 Apr 7;30(4):e14706. doi: 10.1111/cns.14706 (PMC10999557; doi:10.1111/cns.14706)
Supplement: Supplementary file 3 — Table S3 [file CNS-30-e14706-s006.doc]

**Table S3**. Differences in brain regions between the SCI and NC groups, without considering confounding factors.

This report is based on CUI Xu's xjview. (http://www.alivelearn.net/xjview/)

Revised by YAN Chao-Gan and ZHU Wei-Xuan 20091108: suitable for different Cluster Connectivity Criterion: surface connected, edge connected, corner connected.

Number of clusters found: 3

----------------------

Cluster 1

Number of voxels: 26

Peak MNI coordinate: 9 -9 -12

Peak MNI coordinate region: // Right Brainstem // Midbrain // undefined // undefined // undefined // undefined

Peak intensity: -5.3517

# voxels structure

26 --TOTAL # VOXELS--

12 Right Brainstem

12 Midbrain

6 Right Cerebrum

5 Gray Matter

4 Parahippocampa Gyrus

4 brodmann area 34

4 Limbic Lobe

3 ParaHippocampal_R (aal)

1 Mammillary Body

1 Hippocampus_R (aal)

----------------------

Cluster 2

Number of voxels: 21

Peak MNI coordinate: -33 -57 -3

Peak MNI coordinate region: // Left Cerebrum // Temporal Lobe // Sub-Gyral // White Matter // undefined // undefined

Peak intensity: -4.8841

# voxels structure

21 --TOTAL # VOXELS--

21 Left Cerebrum

19 White Matter

15 Occipital Lobe

15 Sub-Gyral

12 Fusiform_L (aal)

5 Temporal Lobe

3 Lingual_L (aal)

3 Fusiform Gyrus

2 Gray Matter

2 brodmann area 19

2 Lingual Gyrus

1 Parahippocampa Gyrus

1 Limbic Lobe

----------------------

Cluster 3

Number of voxels: 27

Peak MNI coordinate: 12 -51 75

Peak MNI coordinate region: // undefined // undefined // undefined // undefined // undefined // Postcentral_R (aal)

Peak intensity: -3.9316

# voxels structure

27 --TOTAL # VOXELS--

13 Right Cerebrum

12 Paracentral_Lobule_R (aal)

12 Parietal Lobe

11 Gray Matter

10 Paracentral Lobule

8 Postcentral Gyrus

8 Precuneus_R (aal)

6 Frontal Lobe

5 brodmann area 4

5 Left Cerebrum

3 brodmann area 7

2 White Matter

2 Postcentral_R (aal)

2 brodmann area 6

2 Paracentral_Lobule_L (aal)

2 Inter-Hemispheric

1 brodmann area 5

1 Precuneus_L (aal)

>>
