# Supplementary material for: Spontaneous brain activity in the hippocampal regions could characterize cognitive impairment in patients with Parkinson's disease
Source: CNS Neurosci Ther. 2024 Apr 7;30(4):e14706. doi: 10.1111/cns.14706 (PMC10999557; doi:10.1111/cns.14706)
Supplement: Supplementary file 4 — Table S4 [file CNS-30-e14706-s002.doc]

**Table S4**. Differences in brain regions between the SCI and NC groups, considering confounding factors.

This report is based on CUI Xu's xjview. (http://www.alivelearn.net/xjview/)

Revised by YAN Chao-Gan and ZHU Wei-Xuan 20091108: suitable for different Cluster Connectivity Criterion: surface connected, edge connected, corner connected.

Number of clusters found: 2

----------------------

Cluster 1

Number of voxels: 21

Peak MNI coordinate: 9 -9 -12

Peak MNI coordinate region: // Right Brainstem // Midbrain // undefined // undefined // undefined // undefined

Peak intensity: -5.0327

# voxels structure

21 --TOTAL # VOXELS--

9 Right Brainstem

9 Midbrain

5 Right Cerebrum

2 Parahippocampa Gyrus

2 Limbic Lobe

2 Gray Matter

2 brodmann area 34

2 ParaHippocampal_R (aal)

1 White Matter

1 Extra-Nuclear

1 Sub-lobar

----------------------

Cluster 2

Number of voxels: 19

Peak MNI coordinate: -33 -54 -3

Peak MNI coordinate region: // Left Cerebrum // Temporal Lobe // Sub-Gyral // White Matter // undefined // undefined

Peak intensity: -4.7214

# voxels structure

19 --TOTAL # VOXELS--

19 Left Cerebrum

17 White Matter

13 Sub-Gyral

12 Occipital Lobe

8 Fusiform_L (aal)

5 Temporal Lobe

2 Sub-lobar

2 Fusiform Gyrus

2 Cerebro-Spinal Fluid

2 Lateral Ventricle

1 Lingual Gyrus

1 Lingual_L (aal)

1 Middle Occipital Gyrus

>>
