# Supplementary material for: Spontaneous brain activity in the hippocampal regions could characterize cognitive impairment in patients with Parkinson's disease
Source: CNS Neurosci Ther. 2024 Apr 7;30(4):e14706. doi: 10.1111/cns.14706 (PMC10999557; doi:10.1111/cns.14706)
Supplement: Supplementary file 5 — Table S5 [file CNS-30-e14706-s004.doc]

**Table S5**. Differential brain regions in the SCI and MCI groups, without correction for confounders.

This report is based on CUI Xu's xjview. (http://www.alivelearn.net/xjview/)

Revised by YAN Chao-Gan and ZHU Wei-Xuan 20091108: suitable for different Cluster Connectivity Criterion: surface connected, edge connected, corner connected.

Number of clusters found: 3

----------------------

Cluster 1

Number of voxels: 45

Peak MNI coordinate: 9 -9 -15

Peak MNI coordinate region: // Right Brainstem // Midbrain // undefined // undefined // undefined // undefined

Peak intensity: -4.4678

# voxels structure

45 --TOTAL # VOXELS--

19 Right Cerebrum

15 Parahippocampa Gyrus

15 Limbic Lobe

15 Midbrain

13 Gray Matter

8 brodmann area 34

8 Left Brainstem

7 ParaHippocampal_R (aal)

7 Amygdala_R (aal)

7 Right Brainstem

5 Amygdala

2 Frontal Lobe

2 Subcallosal Gyrus

1 Hippocampus_R (aal)

----------------------

Cluster 2

Number of voxels: 26

Peak MNI coordinate: 18 -54 18

Peak MNI coordinate region: // Right Cerebrum // Sub-lobar // Extra-Nuclear // White Matter // undefined // Precuneus_R (aal)

Peak intensity: -4.5506

# voxels structure

26 --TOTAL # VOXELS--

26 Right Cerebrum

20 Precuneus_R (aal)

20 White Matter

9 Parietal Lobe

8 Sub-Gyral

7 Extra-Nuclear

7 Sub-lobar

6 Gray Matter

6 Calcarine_R (aal)

6 brodmann area 31

6 Precuneus

5 Limbic Lobe

3 Occipital Lobe

3 Cingulate Gyrus

2 Temporal Lobe

2 Posterior Cingulate

----------------------

Cluster 3

Number of voxels: 29

Peak MNI coordinate: -3 -60 57

Peak MNI coordinate region: // Left Cerebrum // Parietal Lobe // Precuneus // White Matter // undefined // Precuneus_L (aal)

Peak intensity: -4.7584

# voxels structure

29 --TOTAL # VOXELS--

27 Precuneus

27 Parietal Lobe

24 Precuneus_L (aal)

21 Left Cerebrum

13 brodmann area 7

13 Gray Matter

6 Right Cerebrum

6 White Matter

5 Precuneus_R (aal)

2 Inter-Hemispheric

>>
