# Supplementary material for: Spontaneous brain activity in the hippocampal regions could characterize cognitive impairment in patients with Parkinson's disease
Source: CNS Neurosci Ther. 2024 Apr 7;30(4):e14706. doi: 10.1111/cns.14706 (PMC10999557; doi:10.1111/cns.14706)
Supplement: Supplementary file 6 — Table S6 [file CNS-30-e14706-s003.doc]

**Table S6**. Differential brain regions in the SCI and MCI groups, after correction for confounders.

This report is based on CUI Xu's xjview. (http://www.alivelearn.net/xjview/)

Revised by YAN Chao-Gan and ZHU Wei-Xuan 20091108: suitable for different Cluster Connectivity Criterion: surface connected, edge connected, corner connected.

Number of clusters found: 3

----------------------

Cluster 1

Number of voxels: 20

Peak MNI coordinate: 9 -9 -15

Peak MNI coordinate region: // Right Brainstem // Midbrain // undefined // undefined // undefined // undefined

Peak intensity: -4.4092

# voxels structure

20 --TOTAL # VOXELS--

8 Midbrain

5 Right Cerebrum

5 Right Brainstem

3 Limbic Lobe

3 Left Brainstem

3 Parahippocampa Gyrus

2 Gray Matter

2 brodmann area 34

2 ParaHippocampal_R (aal)

----------------------

Cluster 2

Number of voxels: 21

Peak MNI coordinate: 18 -54 18

Peak MNI coordinate region: // Right Cerebrum // Sub-lobar // Extra-Nuclear // White Matter // undefined // Precuneus_R (aal)

Peak intensity: -5.1246

# voxels structure

21 --TOTAL # VOXELS--

21 Right Cerebrum

19 White Matter

14 Precuneus_R (aal)

10 Sub-Gyral

8 Parietal Lobe

7 Sub-lobar

7 Calcarine_R (aal)

7 Extra-Nuclear

4 Temporal Lobe

3 Precuneus

2 brodmann area 31

2 Gray Matter

1 Occipital Lobe

1 Limbic Lobe

1 Posterior Cingulate

----------------------

Cluster 3

Number of voxels: 20

Peak MNI coordinate: 33 -12 60

Peak MNI coordinate region: // Right Cerebrum // Frontal Lobe // Precentral Gyrus // Gray Matter // brodmann area 6 // Precentral_R (aal)

Peak intensity: 4.0843

# voxels structure

20 --TOTAL # VOXELS--

20 Frontal Lobe

20 Right Cerebrum

19 Precentral_R (aal)

19 Precentral Gyrus

15 Gray Matter

15 brodmann area 6

5 White Matter

1 Frontal_Sup_R (aal)

1 Middle Frontal Gyrus

>>
