# Supplementary material for: Spontaneous brain activity in the hippocampal regions could characterize cognitive impairment in patients with Parkinson's disease
Source: CNS Neurosci Ther. 2024 Apr 7;30(4):e14706. doi: 10.1111/cns.14706 (PMC10999557; doi:10.1111/cns.14706)
Supplement: Supplementary file 7 — Table S7 [file CNS-30-e14706-s005.doc]

**Table S7**. Without considering confounding factors, the hippocampal subregions characterizing cognition.

This report is based on CUI Xu's xjview. (http://www.alivelearn.net/xjview/)

Revised by YAN Chao-Gan and ZHU Wei-Xuan 20091108: suitable for different Cluster Connectivity Criterion: surface connected, edge connected, corner connected.

Number of clusters found: 6

----------------------

Cluster 1

Number of voxels: 4

Peak MNI coordinate: 27 6 -33

Peak MNI coordinate region: // Right Cerebrum // Limbic Lobe // Uncus // White Matter // undefined // ParaHippocampal_R (aal)

Peak intensity: 0.38189

# voxels structure

4 --TOTAL # VOXELS--

4 Limbic Lobe

4 ParaHippocampal_R (aal)

4 Right Cerebrum

4 Uncus

4 White Matter

----------------------

Cluster 2

Number of voxels: 2

Peak MNI coordinate: -27 -12 -27

Peak MNI coordinate region: // Left Cerebrum // Limbic Lobe // Parahippocampa Gyrus // White Matter // undefined // ParaHippocampal_L (aal)

Peak intensity: 0.40127

# voxels structure

2 --TOTAL # VOXELS--

2 Left Cerebrum

2 Limbic Lobe

2 ParaHippocampal_L (aal)

2 Parahippocampa Gyrus

1 Gray Matter

1 White Matter

1 brodmann area 35

----------------------

Cluster 3

Number of voxels: 3

Peak MNI coordinate: 15 -6 -21

Peak MNI coordinate region: // Right Cerebrum // Limbic Lobe // Parahippocampa Gyrus // Gray Matter // brodmann area 34 // ParaHippocampal_R (aal)

Peak intensity: 0.44689

# voxels structure

3 --TOTAL # VOXELS--

3 Gray Matter

3 Limbic Lobe

3 ParaHippocampal_R (aal)

3 Parahippocampa Gyrus

3 Right Cerebrum

2 brodmann area 34

1 Amygdala

----------------------

Cluster 4

Number of voxels: 1

Peak MNI coordinate: -24 -30 -15

Peak MNI coordinate region: // Left Cerebrum // Limbic Lobe // Parahippocampa Gyrus // Gray Matter // brodmann area 36 // ParaHippocampal_L (aal)

Peak intensity: 0.35403

# voxels structure

1 --TOTAL # VOXELS--

1 Gray Matter

1 Left Cerebrum

1 Limbic Lobe

1 ParaHippocampal_L (aal)

1 Parahippocampa Gyrus

1 brodmann area 36

----------------------

Cluster 5

Number of voxels: 1

Peak MNI coordinate: 39 -18 -15

Peak MNI coordinate region: // Right Cerebrum // Temporal Lobe // Sub-Gyral // White Matter // undefined // Hippocampus_R (aal)

Peak intensity: 0.34712

# voxels structure

1 --TOTAL # VOXELS--

1 Hippocampus_R (aal)

1 Right Cerebrum

1 Sub-Gyral

1 Temporal Lobe

1 White Matter

----------------------

Cluster 6

Number of voxels: 3

Peak MNI coordinate: 15 -27 -6

Peak MNI coordinate region: // Right Brainstem // Midbrain // undefined // undefined // undefined // undefined

Peak intensity: 0.37573

# voxels structure

3 --TOTAL # VOXELS--

3 Right Brainstem

3 Midbrain

1 Hippocampus_R (aal)

>>
