# Supplementary material for: Spontaneous brain activity in the hippocampal regions could characterize cognitive impairment in patients with Parkinson's disease
Source: CNS Neurosci Ther. 2024 Apr 7;30(4):e14706. doi: 10.1111/cns.14706 (PMC10999557; doi:10.1111/cns.14706)
Supplement: Supplementary file 8 — Table S8 [file CNS-30-e14706-s008.doc]

**Table S8**. Considering confounding factors, the hippocampal subregions characterizing cognition.

This report is based on CUI Xu's xjview. (http://www.alivelearn.net/xjview/)

Revised by YAN Chao-Gan and ZHU Wei-Xuan 20091108: suitable for different Cluster Connectivity Criterion: surface connected, edge connected, corner connected.

Number of clusters found: 9

----------------------

Cluster 1

Number of voxels: 2

Peak MNI coordinate: 27 6 -33

Peak MNI coordinate region: // Right Cerebrum // Limbic Lobe // Uncus // White Matter // undefined // ParaHippocampal_R (aal)

Peak intensity: 0.40209

# voxels structure

2 --TOTAL # VOXELS--

2 Limbic Lobe

2 ParaHippocampal_R (aal)

2 Right Cerebrum

2 Uncus

2 White Matter

----------------------

Cluster 2

Number of voxels: 1

Peak MNI coordinate: -27 -12 -27

Peak MNI coordinate region: // Left Cerebrum // Limbic Lobe // Parahippocampa Gyrus // White Matter // undefined // ParaHippocampal_L (aal)

Peak intensity: 0.35175

# voxels structure

1 --TOTAL # VOXELS--

1 Left Cerebrum

1 Limbic Lobe

1 ParaHippocampal_L (aal)

1 Parahippocampa Gyrus

1 White Matter

----------------------

Cluster 3

Number of voxels: 1

Peak MNI coordinate: 30 -9 -24

Peak MNI coordinate region: // Right Cerebrum // Limbic Lobe // Parahippocampa Gyrus // Gray Matter // Hippocampus // Hippocampus_R (aal)

Peak intensity: 0.35473

# voxels structure

1 --TOTAL # VOXELS--

1 Gray Matter

1 Hippocampus

1 Hippocampus_R (aal)

1 Limbic Lobe

1 Parahippocampa Gyrus

1 Right Cerebrum

----------------------

Cluster 4

Number of voxels: 4

Peak MNI coordinate: 15 -6 -21

Peak MNI coordinate region: // Right Cerebrum // Limbic Lobe // Parahippocampa Gyrus // Gray Matter // brodmann area 34 // ParaHippocampal_R (aal)

Peak intensity: 0.4908

# voxels structure

4 --TOTAL # VOXELS--

4 Gray Matter

4 Limbic Lobe

4 ParaHippocampal_R (aal)

4 Parahippocampa Gyrus

4 Right Cerebrum

3 brodmann area 34

1 Amygdala

----------------------

Cluster 5

Number of voxels: 1

Peak MNI coordinate: 39 -18 -15

Peak MNI coordinate region: // Right Cerebrum // Temporal Lobe // Sub-Gyral // White Matter // undefined // Hippocampus_R (aal)

Peak intensity: 0.43687

# voxels structure

1 --TOTAL # VOXELS--

1 Hippocampus_R (aal)

1 Right Cerebrum

1 Sub-Gyral

1 Temporal Lobe

1 White Matter

----------------------

Cluster 6

Number of voxels: 1

Peak MNI coordinate: 15 -36 -9

Peak MNI coordinate region: // Right Cerebellum // Cerebellum Anterior Lobe // Culmen // undefined // undefined // ParaHippocampal_R (aal)

Peak intensity: -0.36607

# voxels structure

1 --TOTAL # VOXELS--

1 Culmen

1 ParaHippocampal_R (aal)

1 Right Cerebellum

1 Cerebellum Anterior Lobe

----------------------

Cluster 7

Number of voxels: 1

Peak MNI coordinate: 30 -30 -9

Peak MNI coordinate region: // Right Cerebrum // Limbic Lobe // Parahippocampa Gyrus // White Matter // undefined // Hippocampus_R (aal)

Peak intensity: 0.34767

# voxels structure

1 --TOTAL # VOXELS--

1 Hippocampus_R (aal)

1 Limbic Lobe

1 Parahippocampa Gyrus

1 Right Cerebrum

1 White Matter

----------------------

Cluster 8

Number of voxels: 3

Peak MNI coordinate: 15 -30 -3

Peak MNI coordinate region: // Right Brainstem // Midbrain // undefined // undefined // undefined // undefined

Peak intensity: 0.49155

# voxels structure

3 --TOTAL # VOXELS--

3 Right Brainstem

3 Midbrain

1 Hippocampus_R (aal)

----------------------

Cluster 9

Number of voxels: 1

Peak MNI coordinate: -18 -36 6

Peak MNI coordinate region: // Left Cerebrum // Sub-lobar // Extra-Nuclear // White Matter // undefined // Hippocampus_L (aal)

Peak intensity: 0.35444

# voxels structure

1 --TOTAL # VOXELS--

1 Extra-Nuclear

1 Hippocampus_L (aal)

1 Left Cerebrum

1 Sub-lobar

1 White Matter

>>
